# Synthesis and Biological Screening of New Cyano-Substituted Pyrrole Fused (Iso)Quinoline Derivatives

**DOI:** 10.3390/molecules26072066

**Published:** 2021-04-03

**Authors:** Maria Cristina Al-Matarneh, Roxana-Maria Amărandi, Ionel I. Mangalagiu, Ramona Danac

**Affiliations:** 1 Department of Chemistry, Faculty of Chemistry, Alexandru Ioan Cuza University of Iași, 11 Carol I, 700506 Iași, Romania; roxana.amarandi@chem.uaic.ro (R.-M.A.); ionelm@uaic.ro (I.I.M.); 2 “Petru Poni” Institute of Macromolecular Chemistry of Romanian Academy, 41A Grigore Ghica Voda Alley, 700487 Iași, Romania; 3 TRANSCEND Research Center, Regional Institute of Oncology, 2-4 General Henri Mathias Berthelot Street, 700483 Iași, Romania

**Keywords:** 3 + 2 cycloaddition, pyrrolo fused (iso)quinoline, anticancer, tubulin polymerization inhibitor, molecular docking

## Abstract

Several new cyano-substituted derivatives with pyrrolo[1,2-*a*]quinoline and pyrrolo[2,1-*a*]isoquinoline scaffolds were synthesized by the [3 + 2] cycloaddition of (iso)quinolinium ylides to fumaronitrile. The cycloimmonium ylides reacted in situ as 1,3-dipoles with fumaronitrile to selectively form distinct final compounds, depending on the structure of the (iso)quinolinium salt. Eleven compounds were evaluated for their anticancer activity against a panel of 60 human cancer cell lines. The most potent compound **9a** showed a broad spectrum of antiproliferative activity against cancer cell lines representing leukemia, melanoma and cancer of lung, colon, central nervous system, ovary, kidney, breast and prostate cancer. In vitro assays and molecular docking revealed tubulin interaction properties of compound **9a**.

## 1. Introduction

Fused polyazaheterocyclic derivatives are essential structural motifs, being commonly present in natural products and bioactive agents. Due to their wide range of biological activities, they represent one of the most important classes of organic compounds, with extensive applications in medicinal chemistry [1].

Their design, usually based on a combinatorial method having the purpose of assembling different pharmacophores in the same molecular framework, is of considerable interest due to the large variety of such molecules showing promising biological activities. Hybrid drugs are of particular interest since they can potentially eliminate most of the problems encountered with currently used drugs such as toxicity, major side effects and drug resistance [2,3,4].

**Figure 1:**
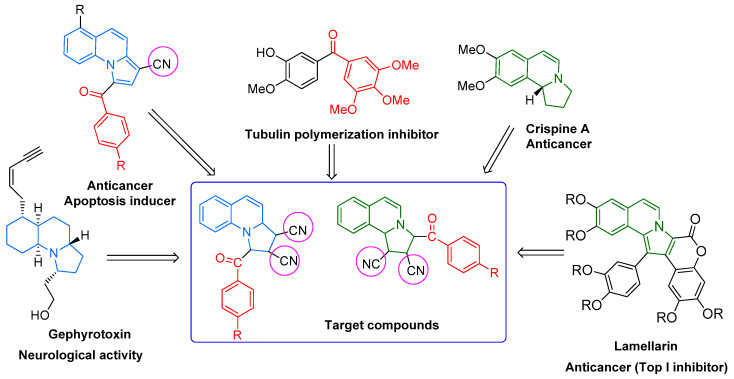
Design in the class of cyano-substituted pyrrolo(iso)quinolines.

Particularly, fused compounds containing quinoline and isoquinoline moieties constitute select classes of compounds, both heterocycles being widely explored for the development of bioactive molecules [3,5,6]. Notably, pyrrolo[2,1-*a*]isoquinoline is a common scaffold in multiple alkaloids (crispines, trolline, lamellarins) having diverse biological action, including antitumor, antibacterial, antifungal and antiviral activities [7,8,9]. For example, lamellarin D, a natural marine alkaloid, is a topoisomerase I inhibitor that induces apoptosis in several cancer cell lines [8,9,10], while crispine A is a potent antiproliferative alkaloid [11] (Figure 1). In addition, the pyrrolo[1,2-*a*]quinoline moiety is present in the natural alkaloid gephyrotoxin, a muscarinic antagonist [12,13], while other pyrrolo[1,2-*a*]quinoline derivatives are known as activators of caspases and inducers of apoptosis by inhibiting tubulin polymerization and effective anticancer agents (Figure 1) [14,15].

Considering the biological relevance of these two important scaffolds and the positive tendency for their further development, pyrrolo(iso)quinoline derivatives have increasingly become targets of interest in the organic synthetic community. Over the past decades, several synthetic methodologies to generate compounds with pyrrolo[2,1-*a*]isoquinoline or pyrrolo[1,2-*a*]quinoline cores have been developed, including the [3 + 2] cycloaddition of the corresponding cycloimmonium ylides to various dipolarophiles, cyclization of lactam derivatives, intramolecular cyclizations, as well as several transition metal catalyzed C-C and C–N bond formation reactions [3,6,16]. It is worth mentioning that the nature and the position of the substituents in the core-containing compounds define their biological profile.

Taking into account the abovementioned details, as well as our ongoing interest in developing new bioactive fused heterocycles [17,18,19,20,21,22], we present herein the design and synthesis of novel pyrrolo[1,2-*a*]quinoline and pyrrolo[2,1-*a*]isoquinoline derivatives which show great promise as anticancer agents. The compounds bear cyano- and 4-substituted phenacyl groups as substituents at the pyrrole ring. The choice of the substituents was based on the observation that both cyano and 4-substituted benzoyl groups were found to be important for the anticancer activity of various bioactive molecules [14,15,23] (Figure 1). The new compounds were evaluated for their anticancer activity by screening against a panel of 60 human tumor cell lines. Moreover, tubulin polymerization assays and molecular docking studies were carried out in order to gain some insights into the mode of action of the tested compounds.

## 2. Results and Discussion

**Scheme 1:**
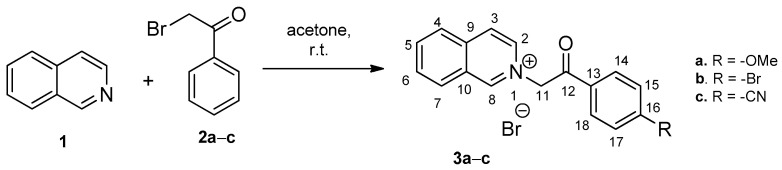
Synthesis of isoquinolin-2-ium monoquaternary salts **3.**

**Scheme 2:**
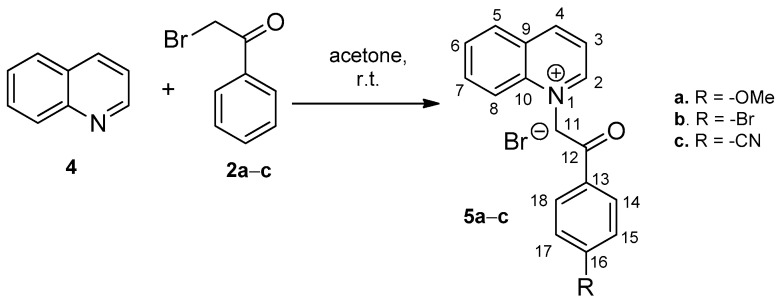
Synthesis of quinolin-1-ium monoquaternary salts **5**.

Pyrrolo[1,2-*a*]quinoline and pyrrolo[2,1-*a*]isoquinoline cores were generated through [3 + 2] cycloaddition reactions that lead to the formation of in situ (iso)quinolinium ylides [24,25,26,27]. Initially, for the introduction of a 4-substituted phenacyl substituent, salts **3a**–**c** and **5a**–**c** were synthesized by the direct *N*-alkylation of isoquinoline and quinoline, respectively, using *para*-substituted 2-bromoacetophenones **2a**–**c** (Scheme 1 and Scheme 2) [28]. The spectral data of salts **2a**–**b** and **3a**–**b** are not reported herein, being in agreement with the ones in the existing literature [29,30].

In the next step, dicyano-substituted fused pyrrole ring derivatives were built through the Huisgen [3 + 2] cycloaddition of the in situ formed ylides corresponding to the salts **3** and **5**, to fumaronitrile.

**Scheme 3:**
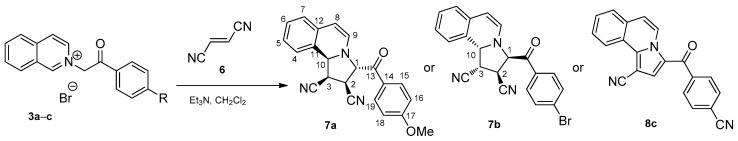
Synthesis of fused compounds **7a**–**b** and **8c**.

Interestingly, following the same experimental protocol consisting in triethylamine treatment of the monoquaternary salt and subsequent cycloaddition to fumaronitrile, we selectively obtained distinct major products in the case of isoquinolin-2-ium salts **3a**–**c** (Scheme 3).

**Scheme 4:**
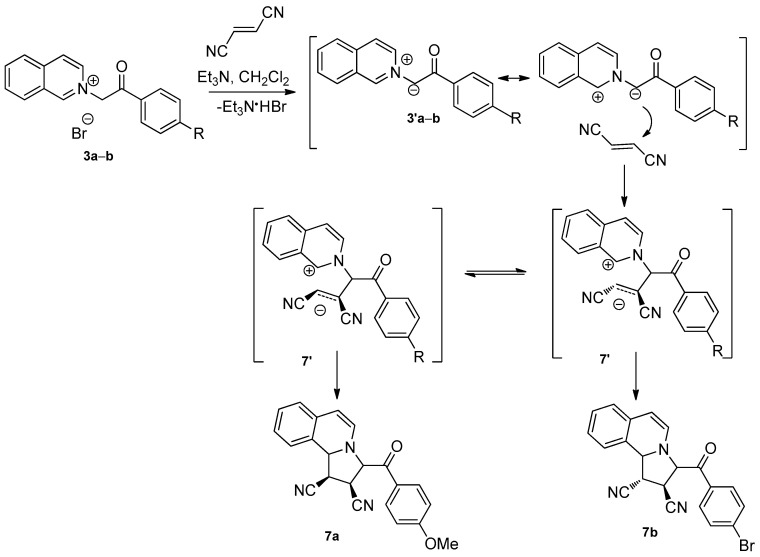
Synthesis pathway and reaction mechanism for the fused isoquinoline derivatives **7a,b**.

**Scheme 5:**
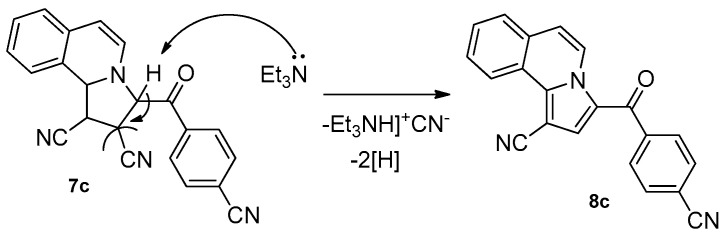
Proposed reaction mechanism for the formation of fused isoquinoline derivative **8c**.

As expected, derivatives with a 1,2,3,10b-tetrahydropyrrolo[2,1-*a*]isoquinoline scaffold were generated in the first step (Scheme 4 and Scheme 5), but in the case of salt **3c** we discovered that the initial fused cycloadduct **7c** underwent an elimination of the cyano group under triethylamine excess, followed by an oxidative aromatization, leading to the more stable compound **8c** (Scheme 5).

The NMR analysis of compounds **7a**–**b** confirmed the formation of one major polychiral racemic with a 1,2,3,10b-tetrahydropyrrolo[2,1-*a*]isoquinoline scaffold, but surprisingly, the geometry of the pyrrole ring is different for compounds **7a**,**b** (Scheme 3).

Thus, in the ^1^H-NMR spectrum of compound **7a**, the signal at δ = 3.79 ppm was assigned to the H3 atom, the most shielded proton of the tetrahydropyrrole cycle. Protons 2 and 10 produce signals at δ = 4.26 ppm and δ = 4.32 ppm, respectively (Table 1). The small coupling for the H1 proton (δ = 6.04 ppm, *J* = 2.5 Hz) suggests a *trans* position relative to proton H2 [20,21,29]. The large values for the coupling constants *J*_2,3_ (8.0 Hz) and *J*_3,10_ (9.5 Hz) suggests that hydrogen atoms H2, H3 and H10 lay on the same side of the tetrahydropyrrole ring, but on the opposite side of H1 (Scheme 3, Table 1).

In the ^1^H-NMR spectrum of compound **7b**, the signal produced by H1 appears as a doublet at weak fields (δ = 6.00 ppm) due to its electron-withdrawing neighbors. The strong coupling for the H1 proton (*J* = 8.00 Hz) suggests a *cis* position relative to proton 2 (Table 1). Also deshielded is proton H10 that furnished a doublet at δ = 5.04 ppm with a large coupling constant (*J* = 6.50 Hz), suggesting a *cis* position relative to proton 3. Protons 2 and 3 supply signals at δ = 4.33 ppm and δ = 4.40 ppm, respectively. The low value for the coupling constant *J*_2,3_ (4.5 Hz) is evidence that hydrogen atoms H2 and H3 are positioned on opposing sides of the tetrahydropyrrole ring. Taken together, these data show that in the case of compound **7b**, hydrogen atoms H1 and H2 are positioned on the same side of the tetrahydropyrrole ring, but on the opposite side of H3 and H10.

In particular, the generated products have a relative configuration of the nitrile groups identical to that observed in the alkene (compound **7b**), as well as reversed (compound **7a**). The loss of the stereo-configuration contributed from the starting material in compound **7a** indicates that a rotation around the NC-C-C-CN bond must have occurred in the course of the reaction. This implies a process that involves the asynchronous formation of new σ bonds and the necessity of the appearance of isomerization of the acyclic zwitterion intermediates type 7′ (Scheme 4) [31,32]. Even if Huisgen [3 + 2] cycloadditions are commonly recognized as taking place in a one-step mechanism, our observations consolidate existing instances of such reactions that can occur in accordance with multi-step mechanisms with the participation of zwitterion intermediates [31,32,33].

**Scheme 6:**
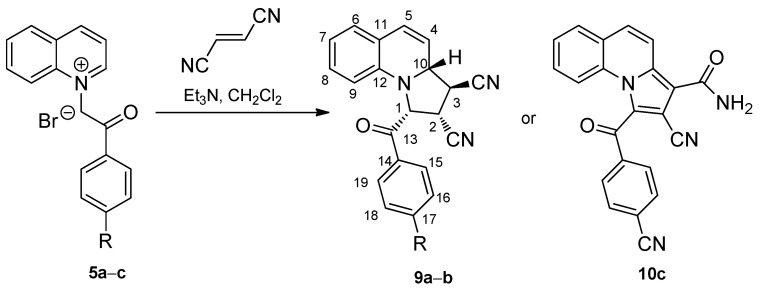
Synthesis of fused quinoline compounds **9a**,**b** and **10c**.

Similarly, by deprotonation of quinolin-1-ium salts **5a**–**c** under triethylamine treatment, the in situ generated resonance stabilized *N*-ylides reacted with fumaronitrile to give tetrahydropyrrolo[1,2-*a*]quinolines **9a**–**b** and pyrrolo[1,2-*a*]quinoline **10c**, respectively (Scheme 6).

The cycloaddition reactions to fumaronitrile occured stereoselectively, a single major polychiral isomer being obtained in case of salts **5a**–**b**. In these cycloaddition reactions, the *trans* stereo-configuration of fumaronitrile was preserved in the pyrrole ring of compounds **9a**–**b** (Scheme 6). The coupling constants of protons H1, H2, H3 and H10 (Scheme 6, Table 2) indicate that protons H1, H2 and H10 lay on the same side of the tetrahydropyrrole ring, but on the opposite side of H3.

The stereoselectivity of the cycloaddition of salt **5c** to fumaronitrile cannot be discussed, as in this case the main product obtained was aromatized 2-cyano-1-(4-cyanobenzoyl)pyrrolo[1,2-*a*]quinoline-3-carboxamide **10c**, due to the hydrolysis of one of the cyano groups at the pyrrole ring.

Eleven compounds were selected for assessment of anticancer activity, which was performed according to the standard protocols of National Cancer Institute (NCI, Rockville, MD, USA). Thus, compounds **3a**–**c**, **5a**–**c**, **7a**–**b**, **8c** and **9a**,**b** and control phenstatin were first tested at a single high dose (10^−5^ M) against a panel of 60 cell lines representing different types of cancers including leukemia, melanoma, lung, colon, CNS, ovarian, renal, prostate and breast cancers [34,35,36]. The results obtained at this stage are summarized in Table 3.

Overall, compounds **9a**,**b**, which possess a 1,2,3,3a-tetrahydropyrrolo[1,2-*a*]quinoline-2,3-dicarbonitrile structure, exhibited good activity against a large number of cancer cells from each panel. In particular, compound **9a** showed a broad spectrum of cytotoxic activity against several of the tested cancer cell lines, the best activity being registered against melanoma MDA-MB-435 cells. Compound **9b** also proved to be cytotoxic against breast cancer MDA-MB-468 cells. The other tested compounds were inactive, except for compound **7a** that selectively inhibited the growth of ovarian cancer OVCAR-8 cells and colon cancer SW-620 cells, as well as compound **8c**, which showed moderate inhibition of several cancer cell lines.

Only compounds which satisfied pre-determined threshold inhibition criteria in a minimum number of cell lines progressed to the full 5-dose assay. The threshold inhibition criteria for progression to the 5-dose screen are used to efficiently capture compounds with anti-proliferative activity based on the careful analysis of in-house screening data available at the Developmental Therapeutics Program at NCI (https://dtp.cancer.gov/discovery_development/nci-60/methodology.htm) (accessed on 2 March 2021).

Due to a broad range of cytostatic and cytotoxic effects, compound **9a** was selected for detailed studies at five different concentrations (0.01, 0.1, 1, 10 and 100 μM) in the 5-dose assay [34,35,36]. The results are represented in terms of response parameter GI_50_, defined as the required molar concentration to inhibit 50% of the growth of cancer cells. Relevant GI_50_ values calculated for compound **9a** against cells of all nine cancer types are summarized in Table 4.

The in vitro screening results revealed that compound **9a** possesses excellent antiproliferative activity, with GI_50_ values ranging from 0.197 to 3.49 μM against 40 cancer cells from all nine sub-panels. Compound **9a** showed the best GI_50_ and total growth inhibition (TGI) values (197 nM and 278 nM, respectively) against HCT-15 colon cancer and SNB-75 CNS cancer.

In order to estimate the toxicity of the most active compound **9a**, we used the web-service Cell-Line Cytotoxicity Predictor (CLC-Pred), which screens for cytotoxicity in silico on a panel of 278 tumour cells and 27 normal human cell lines from different tissues [37]. The predicted toxicity spectrum is represented by a list of activities with probabilities “to be active” *Pa* and “to be inactive” *Pi*. The results (Table 5) predicted cytotoxicity (P_a_ > P_i_ and P_a_ > 0.3) against several cancer cell lines, including two of the tested NCI cell lines: DU-145 and PC-3 (Table 3 and Table 4). The fact that no normal human cell lines appeared on the list could be an indication for a good selectivity of compound 9a against cancer cell lines. However, in vitro testing should be performed in order to confirm these theoretical results.

**Figure 2:**
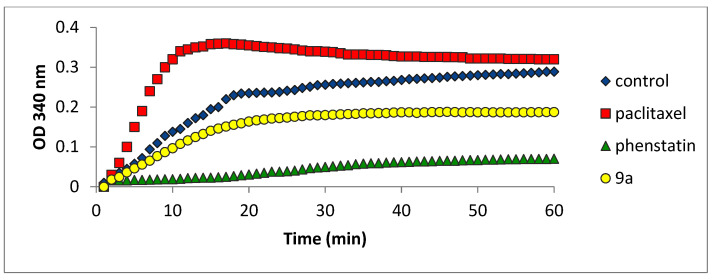
Effect of compound **9a** (10^−5^ M) on microtubule dynamics using paclitaxel (10^−5^ M) as microtubule stabilising control agent and phenstatin (10^−5^ M) as microtubule destabilising control agent.

Cai and collab. previously reported in literature several compounds with pyrrolo[1,2-*a*]quinoline structure and anticancer activity that induced apoptosis by inhibiting tubulin polymerization [14,15]. Therefore, in order to confirm if the observed anticancer activity is related to a microtubule-targeting mechanism, we evaluated the effect of the active compound **9a** on tubulin polymerization (Figure 2). Paclitaxel (as tubulin stabiliser) and phenstatin (as tubulin polymerisation inhibitor) were used as positive controls.

As can be seen in Figure 2, paclitaxel was found to stimulate tubulin polymerisation, while phenstatin and compound **9a** inhibited tubulin polymerisation. However, the tubulin polymerization inhibition potency of compound **9a** was much lower than the one showed by phenstatin, this fact suggesting that other possible mechanisms could be considered for the observed anticancer activity of compound **9a**.

In order to gain more insights regarding a possible interaction of the anticancer tested compounds with tubulin, we carried out the molecular docking of compounds **3a**–**c**, **5a**–**c**, **7a**,**b**, **8c** and **9a**,**b** in the colchicine binding site of tubulin.

Simple isoquinoline and quinoline analogues were accommodated in the colchicine binding site in two distinct orientation modes, dependent on base ring structure. Isoquinolines **3a**–**c** roughly occupied the same space as phenstatin in the tubulin binding site regardless of second ring substituents, although in a flipped manner when compared to other heterocyclic compounds with pronounced anticancer activity [19]. Thus, the isoquinoline moiety overlapped with the trimethoxysubstituted ring of phenstatin, being stabilized exclusively through hydrophobic interactions with side chains mainly in the β subunit (βCys241, βLeu255, βLeu242, βAla250, βLeu248, αAla180). This has been previously seen with other heterocyclic compounds possessing low anticancer activity, yet ability to inhibit tubulin polymerization in vitro [17]. Quinolines **5a**–**c** spanned the dimer interface by anchoring to βAsn258 and engaging in hydrophobic contacts with fewer β subunit side chains when compared to more active compounds (Table 6). The quinoline moiety was not buried deep in the binding site, as seen for isoquinoline analogues, and lacked any interaction with βCys241, which is known to be important for the activity of other antitumor compounds which bind to the colchicine binding site [38,39]. However, the H-bond between the central carbonyl and βAsn258 permitted a favorable geometry for π-σ stacking between the quinoline ring and βLeu248, which has also been seen for some anticancer triazole derivatives [40]. The second ring was oriented toward the α subunit, and in the case of compound **5c**, the best scoring quinoline derivative, engaged in polar contacts with αTyr224 and αGln11 through its cyano substituent. Compound **3c** also had a conformation which formed hydrogen bonds with αTyr224 and αGln11 through its cyano substituent, but of lower binding energy (not shown), indicating that such a binding orientation could also be possible for isoquinoline derivatives, provided the substituents are adequate for hydrogen bond formation.

The slightly more active cycloadducts **7a**,**b** and **8c** were accommodated in the binding pocket similar to quinolines **5a**–**c** rather than parent isoquinolines **3a**–**c**, and were stabilized mostly by hydrophobic contacts with aminoacids in the β subunit, with the exception of **8c**, which established additional contacts with αTyr224 and αGln11 through its cyano substituent, similar to **5c**, having one of the lowest binding energies of all docked compounds (Table 6).

Interestingly, compounds **9a**,**b** had overlapping poses and very good binding energies for two distinct conformations, the first of which are similar to other previously described active phenstatin analogues [18] (lowest binding energy −9.8 kcal/mol and −10.2 kcal/mol, respectively), stabilized mainly by hydrophobic contacts with βLeu242, βLeu248, βLeu255 and an amide-pi stacking interaction between the quinoline moiety and the backbone NH of βLys352. The other conformations were slightly less energetically favorable (−9.4 kcal/mol and −9.1 kcal/mol, respectively, not shown), were flipped and extended toward the GTP molecule with the substituted benzoyl moiety, being stabilized through extensive hydrophobic contacts with βCys241, βLeu248, βAla250, βLeu255 and a hydrogen bond interaction between the carbonyl oxygen and the sidechain of βAsn258, similar to what was observed for the simpler inactive quinoline analogues **5a**–**c** and compounds **7a**,**b** and **8c**. Thus, it is possible that the stability of two such distinct conformations in the binding site confers the pyrrolo[1,2-*a*]quinoline derivatives a more pronounced anticancer activity when compared to their pyrrolo[2,1-*a*]isoquinoline analogues, and at the same time could explain the more pronounced anticancer activity of pyrrolo[2,1-*a*]quinolines when compared to simple quinoline analogues. However, the stability of these docked conformations should be investigated through subsequent molecular dynamics experiments. For docking protocol validation, we obtained a RMSD value of 0.086 Å between the co-crystallized conformation and best scoring pose of re-docked colchicine. Generally, a RMSD value below 2 Å (the average resolution of a crystal structure) is considered acceptable [41].

## 3. Materials and Methods

### 3.1. Chemistry

All commercially available reagents and solvents employed were used without further purification. Melting points were recorded on an Optronic Melting Point Meter KSP1 (A. Krüss, Hamburg, Germany) and are uncorrected. Analytical thin-layer chromatography was performed with commercial silica gel plates 60 F254 (Merck, Darmstadt, Germany) and visualized with UV light (λ_max_ = 254 or 365 nm). The NMR spectra were recorded on an Avance III 500 MHz spectrometer (Bruker, Vienna, Austria) operating at 500 MHz for ^1^H and 125 MHz for ^13^C. Chemical shifts (δ) were reported in part per million (ppm) and coupling constants (*J*) in Hz. The following abbreviations were used to designate chemical shift multiplicities: s = singlet, d = doublet, t = triplet, q = quartet, m = multiplet, bs = broad singlet. Infrared (IR) data were recorded as films on potassium bromide (KBr) pellets on a Prestige 8400s FT-IR spectrophotometer (Shimadzu, Kyoto, Japan) or a 660 *plus* FTIR spectrophotometer (Jasco, Tokyo, Japan). Analyses indicated by the symbols of the elements or functions were within ± 0.4% of the theoretical values.

#### 3.1.1. General Procedure for Synthesis of Isoquinolinium and Quinolinium Salts **3** and **5**

One mmol of isoquinoline **1** or quinoline **4** was dissolved in 5 mL acetone. Then, 1.1 mmol of reactive halide **2a**–**c** was added and the resultant mixture was stirred at room temperature (rt) for 24 h. The precipitate formed was filtered off and washed with acetone to give the desired product.

#### 3.1.2. General Procedure for Synthesis of Isoquinoline and Quinoline Derivatives **7**, **8**, **9** and **10**

The cycloimmonium salt (1 mmol, 1 equiv.) and dipolarophile (fumaronitrile,1.1 mmol, 1.1 equiv), were added to 5 mL dichloromethane and the obtained suspension was stirred at rt under N_2_ atmosphere. Triethylamine (TEA) (3 mmol, 3 equiv.) was added drop-wise over 1 h (magnetic stirring) and the resulting mixture was further stirred for 24 h at rt. Methanol (5 mL) was added and the resulting mixture was kept for 24 h without stirring. The formed precipitate was collected by filtration to give a solid which was washed with methanol. The product was crystallized from dichloromethane/methanol (1/1, *v*/*v*).

#### 3.1.3. Spectral Data

##### 2-(2-(4-Cyanophenyl)-2-oxoethyl)isoquinolin-2-ium bromide (**3c**)

Brown powder; yield: 92%; mp 247–249 °C; IR (KBr), ν_max_ 3019, 2977, 2871, 2226, 1689, 1595, 1400, 1230, 1169, 1101, 990, 820 cm^−1^; ^1^H-NMR (DMSO-*d*_6_) *δ*: 6.75 (2H, s, H-11), 8.11 (1H, t, *J* = 7.5 Hz, H-6), 8.18 (2H, d, *J* = 8.5 Hz, H-15, H-17), 8.27 (2H, d, *J* = 8.5 Hz, H-14, H-18), 8.33 (1H, t, *J* = 7.5 Hz, H-5), 8.43 (1H, d, *J* = 8.0 Hz, H-4), 8.56 (1H, d, *J* = 8.0 Hz, H-7), 8.72 (1H, d, *J* = 7.0 Hz, H-3), 8.78 (1H, d, *J* = 7.0 Hz, H-2), 10.09 (1H, s, H-8); ^13^C-NMR (DMSO-*d*_6_) *δ*: 66.3 (C-11), 116.3 (C-16), 118.0 (CN), 125.5 (C-3), 126.8 (C-10), 127.5 (C-4), 129.0 (C-14, C-18), 130.7 (C-7), 131.5 (C-6), 133.2 (C-15, C-17), 136.3 (C-2), 136.9 (C-13), 137.3 (C-9), 137.6 (C-5), 151.7 (C-8), 190.6 (C-12); Anal. Calcd. for C_18_H_13_BrN_2_O_2_ C, 61.21; H, 3.71; N, 7.93. Found C, 61.2; H, 4.45; N, 7.95.

##### 1-(2-(4-cyanophenyl)-2-oxoethyl)quinolin-1-ium bromide **5c**

Pink powder; yield: 92%; mp 264–265 °C; IR (KBr), ν_max_ 3018, 2979, 2871, 2228, 1691, 1595, 1402, 1228, 1167, 1103, 991, 823 cm^−1^; ^1^H-NMR (DMSO-*d*_6_) *δ*: 7.08 (2H, s, H-11), 8.07 (1H, t, *J* = 8.0 Hz, H-6), 8.19 (2H, d, *J* = 8.5 Hz, H-15, H-17), 8.23 (1H, t, *J* = 8.0 Hz, H-7), 8.30 (2H, d, *J* = 8.0 Hz, H-14, H-18), 8.34 (1H, dd, *J* = 8.5; 6.0 Hz, H-3), 8.53 (1H, d, *J* = 9.0 Hz, H-5), 8.56 (1H, d, *J* = 8.0 Hz, H-8), 9.48 (1H, d, *J* = 8.0 Hz, H-4), 9.55 (1H, d, *J* = 5.5 Hz, H-2); ^13^C-NMR (DMSO-*d*_6_) *δ*: 63.5 (C-11), 116.3 (C-16), 118.0 (CN), 119.3 (C-5), 122.2 (C-3), 129.3 (C-14, C-18), 129.4 (C-9), 130.0 (C-6), 130.6 (C-8), 133.0 (C-15, C-17), 136.0 (C-7), 136.9 (C-13), 138.7 (C-10), 148.8 (C-4), 151.0 (C-2), 190.4 (C-12); Anal. Calcd. for C_18_H_13_BrN_2_O_2_ C, 61.21; H, 3.71; N, 7.93. Found C, 61.19; H, 4.46; N, 7.96.

##### 3-(4-Methoxybenzoyl)-1,2,3,10b-tetrahydropyrrolo[2,1-a]isoquinoline-1,2-dicarbonitrile **7a**

Yellow solid; yield: 57%; mp 200–202 °C; IR (KBr), ν_max_ 2950; 2243, 1681, 1628, 1601, 1315, 1269, 1170, 1150, 772 cm^−1^; ^1^H-NMR (DMSO-*d*_6_) *δ*: 3.79 (1H, dd, *J* = 9.5; 8.0 Hz, H-3), 3.85 (1H, s, OMe), 4.26 (1H, dd, *J* = 7.5; 2.5 Hz, H-2), 4.32 (1H, d, *J* = 9.5 Hz, H-10), 5.64 (1H, d, *J* = 7.5 Hz, H-8), 6.04 (1H, d, *J* = 2.5 Hz, H-1), 6.78 (1H, d, *J* = 7.5 Hz, H-9), 7.06–7.10 (3H, overlapped signals, H-4, H-16, H-18), 7.14–7.18 (2H, overlapped signals, H-6, H-7), 7.03 (1H, dt, *J* = 7.5; 1.0 Hz, H-5), 8.11 (2H, d, *J* = 9.0 Hz, H-15, H-19); ^13^C-NMR (DMSO-*d*_6_) *δ*: 29.9 (C-2), 38.8 (C-3), 55.7 (OMe), 62.7 (C-10), 71.5 (C-1), 102.1 (C-8), 114.1 (C-16, C-18), 118.2 (C-21), 119.2 (C-20), 124.5 (C-4), 126.0 (C-11), 126.1 (C-6), 126.6 (C-14), 126.7 (C-7), 129.2 (C-5), 130.3 (C-12), 131.2 (C-15, C-19), 135.2 (C-9), 163.8 (C-17), 190.7 (C-13); Anal. Calcd. for C_22_H_17_N_3_O_2_ C, 74.35; H, 4.82; N, 11.82. Found C, 74.36; H, 4.80; N, 11.85.

##### 3-(4-Bromobenzoyl)-1,2,3,10b-tetrahydropyrrolo[2,1-a]isoquinoline-1,2-dicarbonitrile **7b**

Yellow powder; yield: 80%; mp 207–209 °C; IR (KBr), ν_max_ 2967; 2245, 1695, 1626, 1573, 1482, 1211, 981, 740 cm^−1^; ^1^H-NMR (DMSO-*d*_6_) *δ*: 4.33 (1H, dd, *J* = 7.5; 4.5 Hz, H-3), 4.40 (1H, dd, *J* = 6.0; 4.5 Hz, H-2), 5.04 (1H, d, *J* = 6.5 Hz, H-10), 5.38 (1H, d, *J* = 7.5 Hz, H-8), 6.00 (1H, d, *J* = 8.0 Hz, H-1), 6.46 (1H, d, *J* = 7.5 Hz, H-9), 7.00 (1H, d, *J* = 7.5 Hz, H-4), 7.00 (1H, t, *J* = 7.5 Hz, H-6), 7.19–7.22 (2H, overlapped signals, H-5, H-7), 7.82 (2H, d, *J* = 8.0 Hz, H-16, H-18), 8.05 (2H, d, *J* = 8.5 Hz, H-15, H-19); ^13^C-NMR (DMSO-*d*_6_) *δ*: 32.5 (C-2), 40.3 (C-3), 61.2 (C-10), 67.8 (C-1), 100.3 (C-8), 117.5 (C-20), 118.1 (C-21), 124.2 (C-4), 125.5 (C-11), 125.8 (C-6), 127.0 (C-7), 128.6 (C-17), 128.8 (C-5), 130.9 (C-15, C-19), 131.8 (C-12), 132.1 (C-16, C-18), 133.3 (C-14), 134.3 (C-9), 193.0 (C-13); Anal. Calcd. for C_21_H_14_BrN_3_O C, 62.39; H, 3.49; N, 10.39. Found C, 62.41; H, 3.48; N, 10.42.

##### 3-(4-cyanobenzoyl)pyrrolo[2,1-a]isoquinoline-1-carbonitrile **8c**

Yellow powder; yield: 65%; mp 205–206 °C; IR (KBr), ν_max_ 3149, 2961, 2221, 1612, 1450, 1343, 1260, 1217, 950, 803 cm^−1^; ^1^H-NMR (CDCl_3_) *δ*: 7.39 (1H, d, *J* = 7.5 Hz, H-8), 7.54 (1H, s, H-2), 7.76–7.78 (2H, overlapped signals, H-5, H-6), 7.85–7.88 (3H, overlapped signals, H-16, H-18, H-7), 7.93 (2H, d, *J* = 8.5 Hz, H-15, H-19), 9.02 (1H, dd, *J* = 8.5; 3.5 Hz, H-4), 9.59 (1H, d, *J* = 7.5 Hz, H-9); ^13^C-NMR (CDCl_3_) *δ*: 86.8 (C-1), 115.8 (C-17), 116.6 (C-8), 116.8 (CN), 118.1 (*p*CN), 123.6 (C-3), 123.9 (C-11), 124.4 (C-4), 125.2 (C-9), 127.5 (C-7), 129.3 (C-5), 129.5 (C-2), 129.6 (C-15, C-19), 130.2 (C-12), 130.6 (C-6), 132.6 (C-16, C-18), 138.6 (C-10), 142.9 (C-14), 183.6 (C-13); Anal. Calcd. for C_21_H_11_N_3_O C, 78.49; H, 3.45; N, 13.08. Found C, 78.50; H, 3.42; N, 13.10.

##### 1-(4-methoxybenzoyl)-1,2,3,3a-tetrahydropyrrolo[1,2-a]quinoline-2,3-dicarbonitrile **9a**

Yellow powder; yield: 74%; mp 203–204 °C; IR (KBr), ν_max_ 2972, 2852, 2255, 1690, 1604, 1486, 1314, 1256, 1164, 745 cm^−1^; ^1^H-NMR (DMSO-*d*_6_) *δ*: 3.91 (1H, s, OMe), 4.21 (1H, d, *J* = 4.0 Hz, H-3), 4.72 (1H, d, *J* = 9.0 Hz, H-2), 5.12 (1H, bs, H-10), 5.74 (1H, dd, *J* = 10.0, 1.5 Hz, H-4), 5.86 (1H, d, *J* = 8.0 Hz, H-9), 5.89 (1H, d, *J* = 9.0 Hz, H-1), 6.58 (1H, t, *J* = 7.5 Hz, H-7), 6.60 (1H, d, *J* = 10.0 Hz, H-5), 6.93 (2H, t, *J* = 8.5 Hz, H-8), 6.96 (1H, d, *J* = 7.5 Hz, H-6), 7.14 (2H, d, *J* = 8.5 Hz, H-16, H-18), 8.20 (2H, d, *J* = 8.5 Hz, H-15, H-19); ^13^C-NMR (DMSO-*d*_6_) *δ*: 33.1 (C-2), 40.3 (C-3), 55.8 (OMe), 61.7 (C-10), 62.0 (C-1), 109.8 (C-9), 114.5 (C-16, C-18), 117.1 (C-20), 117.7 (C-21), 117.8 (C-7), 119.0 (C-11), 119.6 (C-4), 127.2 (C-14), 127.4 (C-6), 128.2 (C-5), 129.6 (C-8), 131.3 (C-15, C-19), 141.0 (C-12), 164.3 (C-17), 193.0 (C-13); Anal. Calcd. for C_22_H_17_N_3_O_2_ C, 74.35; H, 4.82; N, 11.82. Found C, 74.38; H, 4.79; N, 11.84.

##### 1-(4-bromobenzoyl)-1,2,3,3a-tetrahydropyrrolo[1,2-a]quinoline-2,3-dicarbonitrile **9b**

Yellow powder; yield: 72%; mp 199–201 °C; IR (KBr), ν_max_ 3127; 2215, 1626, 1459, 1343, 1232, 973, 799 cm^−1^; ^1^H-NMR (DMSO-*d*_6_) *δ*: 4.22 (1H, d, *J* = 4.0 Hz, H-3), 4.72 (1H, dd, *J* = 9.0; 1.0 Hz, H-2), 5.10 (1H, t, *J* = 2.0 Hz, H-10), 5.74 (1H, dd, *J* = 10.0, 2.0 Hz, H-4), 5.90 (1H, d, *J* = 8.0 Hz, H-9), 5.96 (1H, d, *J* = 9.0 Hz, H-1), 6.58–6.63 (2H, overlapped signals, H-7, H-5), 6.93 (1H, t, *J* = 8.0 Hz, H-8), 6.96 (1H, d, *J* = 7.5 Hz, H-6), 7.86 (2H, d, *J* = 8.5 Hz, H-16, H-18), 8.17 (2H, d, *J* = 8.5 Hz, H-15, H-19); ^13^C-NMR (DMSO-*d*_6_) *δ*: 33.1 (C-2), 40.3 (C-3), 61.7 (C-10), 62.7 (C-1), 110.0 (C-9), 117.1 (C-20), 117.6 (C-21), 118.0 (C-7), 118.9 (C-11), 119.6 (C-4), 127.5 (C-6), 128.2 (C-5), 129.2 (C-17), 129.7 (C-8), 130.8 (C-15, C-19), 132.4 (C-16, C-18), 133.1 (C-14), 140.8 (C-12), 194.2 (C-13); Anal. Calcd. for C_21_H_14_BrN_3_O C, 62.39; H, 3.49; N, 10.39. Found C, 62.40; H, 3.48; N, 10.41.

##### 2-cyano-1-(4-cyanobenzoyl)pyrrolo[1,2-a]quinoline-3-carboxamide **10c**

Dark yellow powder; yield: 69%; mp 202–204 °C; IR (KBr), ν_max_ 3151, 2963, 2222, 1610, 1448, 1346, 1261, 1215, 952, 800 cm^−1^; ^1^H-NMR (DMSO-*d*_6_) *δ*: 7.56 (1H, t, *J* = 7.5 Hz, H-7), 7.77 (1H, t, *J* = 7.5 Hz, H-8), 7.94 (1H, d, *J* = 8.5 Hz, H-6), 8.07 (2H, d, *J* = 8.0 Hz, H-16, H-18), 8.09–8.13 (3H, overlapped signals, H-15, H-19, H-4), 8.23 (1H, d, *J* = 8.5 Hz, H-9), 8.47 (1H, d, *J* = 8.5 Hz, H-5), 9.00 (2H, s, NH2); ^13^C-NMR (DMSO-*d*_6_) *δ*: 99.8 (C-3), 104.1 (C-2), 114.0 (CN), 114.4 (*p*CN), 116.8 (C-4), 118.3 (C-17), 125.8 (C-11, C-1), 126.2 (C-7), 127.8 (C-6), 128.3 (C-9), 129.7 (C-15, C-19), 130.2 (C-8), 132.5 (C-16, C-18), 137.4 (C-5), 140.3 (C-14), 146.8 (C-12), 150.5 (C-10), 161.8 (CONH2), 176.4 (C-13); Anal. Calcd. for C_22_H_12_N_4_O_2_ C, 72.52; H, 3.32; N, 15.38. Found C, 72.55; H, 3.30; N, 15.41.

### 3.2. Anticancer Activity

The compounds were tested against a panel of 60 human cancer cell lines at the National Cancer Institute (Rockville, MD, USA). The cytotoxicity experiments were performed using a 48 h exposure protocol which consisted of a sulforhodamine B assay [34,35,36], briefly described below.

The cell lines are grown in RPMI 1640 medium containing 5% fetal bovine serum and 2 mM l-glutamine. For a typical screening experiment, cells are inoculated into 96 well microtiter plates in 100 µL at plating densities ranging from 5000 to 40,000 cells/well, depending on the doubling time of individual cell lines. The plates are then incubated at 37 °C, 5% CO_2_, 95% air and 100% relative humidity for 24 h prior to addition of compounds. After 24 h, two plates of each cell line are fixed in situ with trichloroacetic acid (TCA), to represent a measurement of the cell population for each cell line at the time of drug addition (Tz). Compounds are solubilized in dimethyl sulfoxide at 400-fold the desired final maximum test concentration and stored frozen prior to use. At the time of drug addition, an aliquot of frozen concentrate is thawed and diluted to twice the desired final maximum test concentration with complete medium containing 50 µg/mL gentamicin. Additional serial dilutions are made to provide a total of five drug concentrations plus control. Aliquots of 100 µL of these different drug dilutions are added to the appropriate microtiter wells already containing 100 µL of medium, resulting in the required final drug concentrations.

Following drug addition, the plates are incubated for an additional 48 h at 37 °C, 5% CO_2_, 95% air, and 100% relative humidity. For adherent cells, the assay is terminated by the addition of cold TCA. Cells are fixed in situ by the gentle addition of 50 µL of cold 50% (*w*/*v*) TCA (final concentration, 10% TCA) and incubated for 60 min at 4 °C. The supernatant is discarded, and the plates are washed five times with tap water and air dried. Sulforhodamine B (SRB) solution (100 µL) at 0.4% (*w*/*v*) in 1% acetic acid is added to each well, and plates are incubated for 10 min at room temperature. After staining, unbound dye is removed by washing five times with 1% acetic acid and the plates are air dried. Bound stain is subsequently solubilized with 10 mM trizma base, and the absorbance is read on an automated plate reader at a wavelength of 515 nm. For suspension cells, the methodology is the same except that the assay is terminated by fixing settled cells at the bottom of the wells by gently adding 50 µL of 80% TCA (final concentration, 16% TCA). Using the seven absorbance measurements [time zero, (Tz), control growth, (C), and test growth in the presence of drug at the five concentration levels (Ti)], the percentage growth is calculated at each of the drug concentrations levels. Percentage growth inhibition (PGI) is calculated as:

(Ti − Tz)/(C − Tz)] × 100 for concentrations for which Ti >/= Tz

[(Ti − Tz)/Tz] × 100 for concentrations for which Ti < Tz.

Three dose response parameters are calculated for each experimental agent. Growth inhibition of 50% (GI_50_) is calculated from [(Ti − Tz)/(C − Tz)] × 100 = 50, which is the drug concentration resulting in a 50% reduction in the net protein increase (as measured by SRB staining) in control cells during the drug incubation. The drug concentration resulting in total growth inhibition (TGI) is calculated from Ti = Tz. The LC_50_ (concentration of drug resulting in a 50% reduction in the measured protein at the end of the drug treatment as compared to that at the beginning) indicating a net loss of cells following treatment is calculated from [(Ti − Tz)/Tz] × 100 = −50. Values are calculated for each of these three parameters if the level of activity is reached; however, if the effect is not reached or is exceeded, the value for that parameter is expressed as greater or less than the maximum or minimum concentration tested.

### 3.3. Tubulin Polymerization Assay

Microtubule assembly was studied using a tubulin polymerization assay kit (Cat. #BK006P, Cytoskeleton Inc., Denver, CO, USA) which was used according to the manufacturer’s instructions [42,43]. The polymerization was monitored using a FLUOstar Omega multi-mode microplate reader (BMG LABTECH, Offenburg, Germany). The final buffer concentrations for tubulin polymerization contained 80 mM PIPES (piperazine-*N*,*N*′-bis(2-ethanesulfonic acid)sequisodium salt), pH = 6.9, 2 mM MgCl_2_, 0.5 mM EGTA (ethylene glycol-bis (β-amino-ethyl ether) *N*,*N*,*N*′,*N*′-tetraacetic acid, 1 mM GTP and 10.2% glycerol. Test compounds were added in one single concentration (10 μM) and then all compounds except the purified tubulin were warmed to 37 °C. The reaction was initiated by the addition of tubulin to a final concentration of 3.0 mg/mL. Paclitaxel and phenstatin were used as positive controls under the same conditions. The absorbance was measured at 340 nm for 1 h at 1 min intervals at 37 °C.

### 3.4. Molecular Modelling

Molecular docking experiments were performed using a previously described protocol [18] with slight modifications. In brief, ligands were built in Avogadro v1.2.0 [44] and energetically optimized in the MMFF94 force field using the Steepest Descent algorithm until reaching a local energy minimum. Docking experiments were performed with Autodock Vina [45], using a 22 × 22 × 22 Å^3^ gridbox centred on the colchicine binding site of the α,β-tubulin heterodimer (PDB: 4O2B) [46]. The x,y,z coordinates of the center of the gridbox were (14.316, 67.184, 43.659). Ten poses were generated for each ligand and the best scoring conformations were selected for discussion. RMSD between re-docked and co-crystallized colchicine ligand was used as quality control for the docking protocol. Visual inspection, molecular graphics and analyses were made in the PyMOL Molecular Graphics System, Version 1.8.2. (Schrödinger, LLC, New York, NY, USA) and Discovery Studio Visualizer Version 20.1.0.19295 (Dassault Systemes, BIOVIA Corp., San Diego, CA, USA).

### 3.5. In Silico Prediction of Cytotoxicity

In silico cytotoxicity for compound **9a** was assessed using CLC-Pred-a web-service which provides a cytotoxicity prediction for chemical compounds in order to estimate the relevance of the their inclusion in experimental screening, based on their structural formula [37]. The prediction is based on Prediction of Activity Spectra for Substances (PASS; http://www.way2drug.com/PASSonline) (accessed on 18 March 2021) and the training set was created based on cytotoxicity data retrieved from ChEMBLdb (https://www.ebi.ac.uk/chembldb/) (accessed on 18 March 2021). The list of predicted activities is arranged in descending order according to *Pa–Pi* values. Thus, the most probable activities are at the top of the list. *Pa* (probability “to be active”) estimates the chance that the studied compound belongs to the subclass of active compounds on that particular cell line (resembles the structures of molecules which are typically included in a subset of “actives” in the PASS training set). *Pi* (probability “to be inactive”) estimates the chance that the studied compound belongs to the subclass of inactive compounds (resembles the structures of molecules which are typically included in a subset of “inactives” in the PASS training set).

## 4. Conclusions

The [3 + 2] cycloaddition of several (iso)quinolinium ylides to fumaronitrile was used for the synthesis of two series of compounds with (tetrahydro)pyrrolo[1,2-*a*]quinoline and (tetrahydro)pyrrolo[2,1-*a*]isoquinoline scaffolds, respectively. During cycloaddition, two types of products were generated, with a relative configuration of the nitrile groups identical to that observed in the parent alkene (compounds **7b**, **8a**,**b**), as well as reversed (compound **7a**). The loss of the stereo-configuration originating from the starting material in compound **7a** implies an asynchronous process of formation of new σ bonds during cycloaddition. Also, aromatization occurred in the case of compounds **8c** and **10c**, accompanied by the loss of one cyano group in the case of product **8c** or hydrolysis of one of the cyano groups in the case of product **10c**. Among the eleven compounds selected and tested against sixty human cancer cell lines at NCI, compound **9a** showed excellent growth inhibition, with GI_50_ values ranging from 0.197 to 3.49 μM against 40 cancer cell lines from all nine NCI sub-panels. The anticancer properties of compound **9a** could be linked to its ability to inhibit tubulin polymerization in vitro by binding the colchicine site of tubulin. The anticancer study showed that the tetrahydropyrrolo[1,2-*a*]quinoline scaffold appears to be more favorable in terms of potential anticancer properties when compared to its isoquinoline analogue. Further derivatization and investigation of this class of compounds are worth conducting for the development of new potential anticancer agents.

## Figures and Tables

**Table 1:** Chemical shifts and coupling constants for protons of new formed pyrrole ring in compounds **7a**,**b**.

Compound	H1 δ(ppm)	*J*_1,2_ (Hz)	H2 δ(ppm)	*J*_2,3_ (Hz)	H3 δ(ppm)	*J*_3,10_ (Hz)	H10 δ(ppm)
**7a**	6.04	2.50	4.26	8.00	3.79	9.50	4.32
**7b**	6.00	8.00	4.33	4.50	4.40	6.50	5.04

**Table 2:** Chemical shifts and coupling constants for protons of new formed pyrrole ring in compounds **9a**,**b**.

Compound	H1 δ(ppm)	*J*_1,2_ (Hz)	H2 δ(ppm)	*J*_2,3_ (Hz)	H3 δ(ppm)	*J*_3,10_ (Hz)	H10 δ(ppm)
**9a**	5.89	9.00	4.72	4.00	4.21	-	5.12
**9b**	5.96	9.00	4.72	4.00	4.22	2.00	5.10

**Table 3:** Results of the in vitro growth inhibition (GI%, 10^−5^ M) caused by compounds **3a**,**b**, **5a–c**, **7a**,**b**, **8c** and **9a**,**b** against human cancer cell lines in the single-dose assay ^a^.

Cell Type	Cell Line	Compound											
		3a	3b	3c	5a	5b	5c	7a	7b	8c	9a	9b	Phenstatin
Leukemia	CCRF-CEM	8	6	2	1	0	0	18	0	0	84	15	94
	K-562	4	20	0	11	17	7	27	0	12	88	76	91
	SR	0	6	0	0	0	3	0	0	25	79	75	93
	HL-60(TB)	0	14	0	2	0	1	6	0	8	100 ^b,d^	6	100 ^b,l^
	MOLT-4	3	7	2	0	0	0	5	0	7	63	8	85
	RPMI-8226	0	6	3	0	1	0	27	0	0	70	6	87
Non-small Cell Lung Cancer	A549/ATCC	0	3	1	2	2	0	13	17	10	70	35	82
	HOP-92	18	31	24	15	24	14	15	5	20	16	45	48
	HOP-62	10	18	9	3	13	0	7	3	66	70	25	77
	NCI-H460	0	3	0	0	0	0	3	13	28	81	4	93
	NCI-H522	8	11	2	8	6	4	29	12	36	100	22	88
Colon Cancer	COLO205	1	18	8	0	0	0	0	0	0	100 ^b,e^	97	58
	HCT-116	3	27	11	4	5	2	14	13	15	85	32	96
	HCT-15	2	5	2	8	1	0	5	4	5	83	77	96
	HT-29	5	33	0	3	2	5	16	14	20	93	63	85
	SW-620	7	13	9	5	8	4	82	0	8	74	34	78
	KM12	6	10	1	1	2	4	2	0	10	94	78	91
CNS Cancer	SF-295	3	17	2	7	5	5	5	4	36	78	11	100 ^b,m^
	SF-539	1	8	5	0	0	2	5	11	52	100 ^b,f^	11	100 ^b,n^
	SNB-75	20	24	30	29	29	17	26	10	67	100 ^b,f^	88	100 ^b,o^
	U251	0	1	2	3	0	0	7	0	15	66	16	79
Melanoma	LOX IMVI	5	14	2	6	7	8	13	8	20	57	22	85
	M14	5	13	8	7	6	5	6	0	3	100 ^b,g^	25	100 ^b,p^
	MDA-MB-435	0	8	2	0	2	0	4	0	0	100 ^b,h^	84	100 ^b,r^
	UACC-62	14	18	0	11	11	13	37	2	35	53	37	55
	SK-MEL-2	0	3	0	0	0	0	12	2	7	66	0	40
	SK-MEL-5	0	2	0	0	1	0	14	10	11	85	39	100 ^b,s^
Ovarian Cancer	OVCAR-3	3	21	0	0	3	0	8	0	8	100 ^b,i^	25	100 ^b,g^
	NCI/ADR-RES	3	6	2	2	0	0	13	6	45	100 ^b,d^	36	100 ^b,t^
	SK-OV-3	3	19	4	0	18	1	7	0	0	45	21	53
	OVCAR-4	3	17	4	0	9	0	38	46	57	54	57	69
	OVCAR-8	3	2	5	2	3	0	100 ^b,c^	6	58	73	10	86
Renal cancer	A498	7	11	56	0	5	3	20	0	5	100 ^b,j^	33	25
	RXF393	1	7	0	0	1	0	23	0	25	100 ^b,k^	25	99
	UO-31	19	29	17	14	20	18	40	1	16	68	52	67
Breast cancer	MCF7	8	14	11	11	17	10	9	8	17	92	87	94
	MDA-MB-468	10	16	10	4	9	15	36	0	23	84	100 ^b,d^	100 ^b,u^
	HS 578T	5	9	6	10	8	5	5	0	50	86	23	71
	BT-549	0	0	5	0	0	0	5	0	47	47	7	88
Prostate cancer	PC-3	11	18	8	10	13	9	16	6	39	71	24	80
	DU-145	0	0	0	0	0	0	0	3	18	76	9	90

^a^ Data obtained from NCI’s in vitro 60 cell one dose screening at 10^−5^ M concentration; ^b^ Cytotoxic effect; Cell growth percent: ^c^ −5; ^d^ −8; ^e^ −6; ^f^ −21; ^g^ −7; ^h^ −48; ^i^ −12; ^j^ −10; ^k^ 22; ^l^ −29; ^m^ −9; ^n^ −22; ^o^ −1; ^p^ −4; ^r^ −41; ^s^ −60; ^t^ −32; ^u^ −14.

**Table 4:** Results of the 5-dose in vitro human cancer cell growth inhibition ^a^ for compound **9a**.

Cell Type	Compound	9a
	Cell Line	GI_50_ (μM) ^b^
Leukemia	HL-60(TB)	1.23
	SR	0.544
	K-562	0.405
Non-small Cell Lung Cancer	NCI-H460	0.749
	NCI-H522	2.45
	A549/ATCC	2.40
Colon Cancer	HCT-116	0.653
	HCT-15	0.197
	HT29	0.844
	SW-620	0.715
	KM12	1.25
Prostate cancer	PC-3	1.45
	DU-145	2.93
Melanoma	SK-MEL-5	3.49
	M14	0.50
	MDA-MB-435	0.335
	UACC-62	0.781
	LOXIMVI	2.10
	MALME-3M	2.67
	Cell line	GI_50_ (μM) ^b^
Ovarian Cancer	OVCAR-3	0.642
	NCI/ADR-RES	0.572
	IGROV1	2.74
	OVCAR-8	3.29
Renal Cancer	CAKI-1	0.460
	A498	0.244
	UO-31	1.95
	ACHN	1.43
	RXF 393	1.52
Breast cancer	MCF7	1.09
	HS 578T	2.10
	MDA-MB-231/ATCC	1.46
	BT-549	1.98
	T-47D	2.39
	MDA-MB-468	2.31
CNS Cancer	SF-295	2.83
	SF-539	1.32
	SNB-75	0.278
	U251	2.81

^a^ Data obtained from from NCI’s in vitro 60 cell 5-dose screening. ^b^ GI_50_—the molar concentration of tested compound causing 50% growth inhibition of tumor cells.

**Table 5:** Results of for the prediction of cytotoxicity of compound **9a**.

P_a_	P_i_	Cell Line	Cell Type
0.698	0.009	DU-145	Prostate carcinoma
0.521	0.004	A-375	Malignant melanoma
0.500	0.029	PC-3	Prostate carcinoma
0.438	0.035	A2058	Melanoma
0.308	0.007	LNCaP	Prostate carcinoma
0.399	0.100	YAPC	Pancreatic carcinoma
0.391	0.125	Hs 683	Oligodendroglioma
0.340	0.080	U-266	Plasma cell myeloma
0.323	0.214	NALM-6	Adult B acute lymphoblastic leukemia
0.329	0.249	MDA-MB-453	Breast adenocarcinoma

**Table 6:** Binding orientation, energy and amino acid contacts for tested compounds, as predicted by molecular docking experiments. For binding orientation, the α,β-tubulin heterodimer is shown as ribbons (red/yellow/green–α subunit helices/sheets/loops; pink/orange/magenta–β subunit helices/sheets/loops); relevant aminoacids, GTP and ligands are represented as sticks; for 2D interaction diagrams, colors are as follows: conventional hydrogen bonds–green, carbon-hydrogen bonds–pale green; hydrophobic interactions–light pink; amide-pi stacking–dark pink; pi-sigma stacking–purple.

Compound	Binding Energy (kcal/mol)	Binding Orientation	2D Interaction Diagram
**Phen**	−7.7	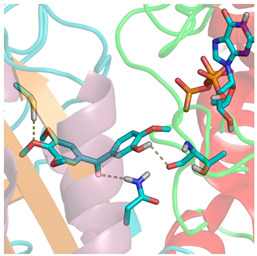	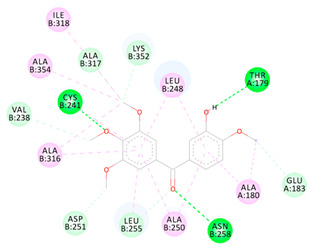
**3a**	−8.7	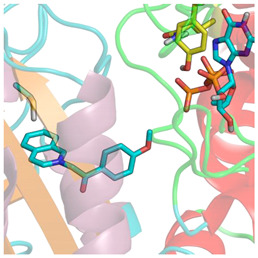	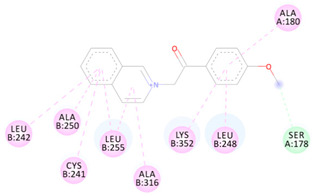
**3b**	−8.6	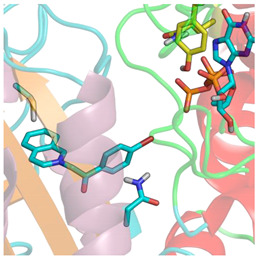	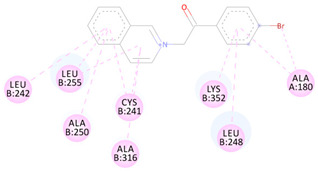
**3c**	−9.2	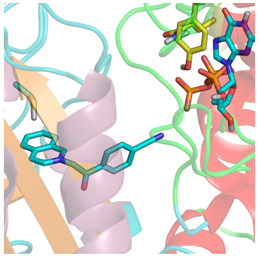	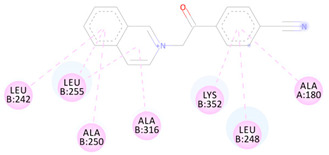
**5a**	−8.6	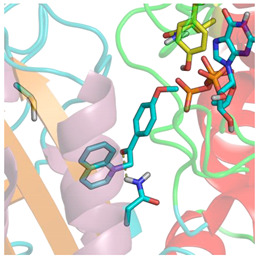	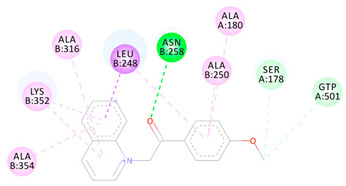
**5b**	−8.6	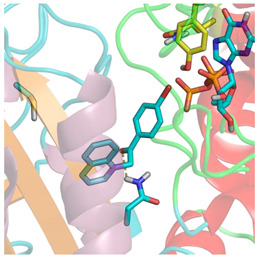	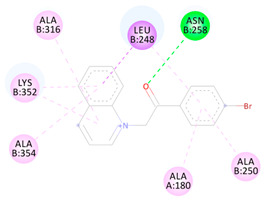
**5c**	−9.3	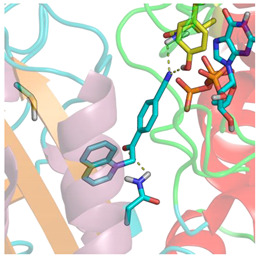	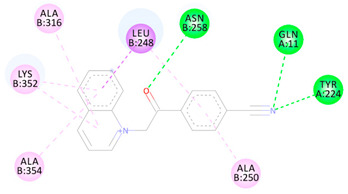
**7a**	−9.7	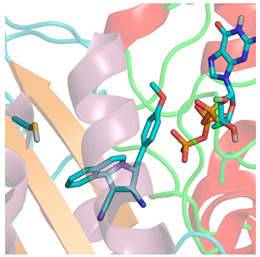	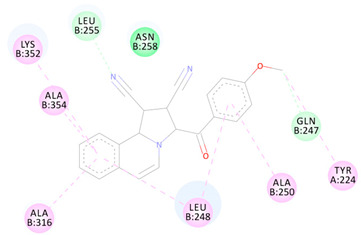
**7b**	−9.4	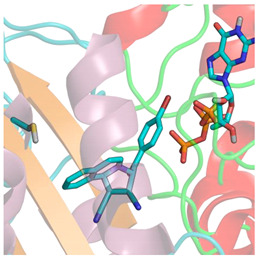	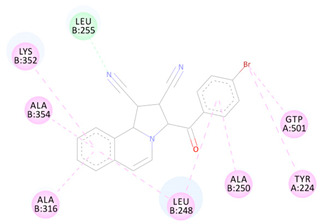
**8c**	−10.8	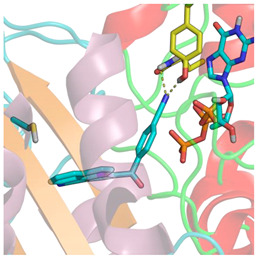	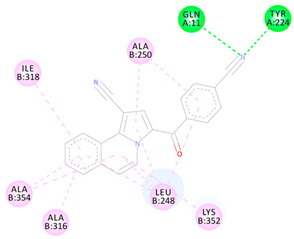
**9a**	−9.8	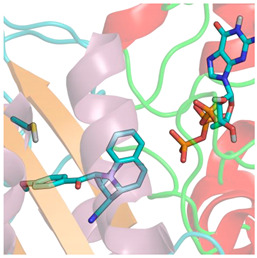	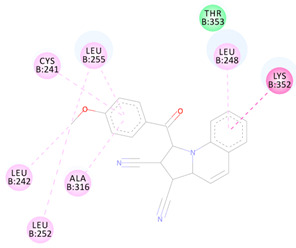
**9b**	−10.1	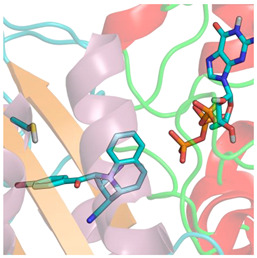	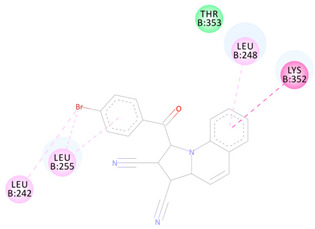

## Data Availability

The data presented in this study are available on request from the corresponding author.

## Sample Availability

Samples of the compounds are available from the authors.
